# An Approach for the Accurate Measurement of Social Morality Levels

**DOI:** 10.1371/journal.pone.0079852

**Published:** 2013-11-27

**Authors:** Haiyan Liu, Xia Chen, Bo Zhang

**Affiliations:** 1 School of Education in Ideology and Politics, China University of Geosciences (Beijing), Beijing, China; 2 College of Information Science and Technology, Beijing Language and Culture University, Beijing; 3 Zhengzhou Normal University, Zhengzhou, China; University of Warwick, United Kingdom

## Abstract

In the social sciences, computer-based modeling has become an increasingly important tool receiving widespread attention. However, the derivation of the quantitative relationships linking individual moral behavior and social morality levels, so as to provide a useful basis for social policy-making, remains a challenge in the scholarly literature today. A quantitative measurement of morality from the perspective of complexity science constitutes an innovative attempt. Based on the NetLogo platform, this article examines the effect of various factors on social morality levels, using agents modeling moral behavior, immoral behavior, and a range of environmental social resources. Threshold values for the various parameters are obtained through sensitivity analysis; and practical solutions are proposed for reversing declines in social morality levels. The results show that: (1) Population size may accelerate or impede the speed with which immoral behavior comes to determine the overall level of social morality, but it has no effect on the level of social morality itself; (2) The impact of rewards and punishment on social morality levels follows the “5∶1 rewards-to-punishment rule,” which is to say that 5 units of rewards have the same effect as 1 unit of punishment; (3) The abundance of public resources is inversely related to the level of social morality; (4) When the cost of population mobility reaches 10% of the total energy level, immoral behavior begins to be suppressed (i.e. the 1/10 moral cost rule). The research approach and methods presented in this paper successfully address the difficulties involved in measuring social morality levels, and promise extensive application potentials.

## Introduction

A refined understanding of morality is the crystallization of human wisdom, and also an embodiment of social civilization and progress. The level of morality is an important measure of the vitality of a society or an era. A review of Chinese history would quickly reveal that the rise and fall of every dynastic house or regime was accompanied by a similar pattern of moral improvement and deterioration – the dates and the dynastic titles may be different, but the evolutionary trajectories were basically the same. This is what we often refer to as the “Law of Dynastic Cycles.” Over the last 30 years, China's rapid development has brought about enormous economic benefits, but that had also been accompanied by deteriorations in social justice, morality, and trust at the same time [1]. In the face of such deteriorations, a great deal of research has been conducted approaching the problem from cultural, institutional, public policy and various other perspectives [2]. However, most of these studies have been of little help to policymakers in assessing the likely effectiveness a new policy during its formulation, and as a result policies continue to be made through costly trial-and-error. This inefficient policy-making process has proved enduring.

Morality is sustained through public opinion, traditions and people's inner beliefs; it is the sum total of the norms and evaluative principles by which judgments of good and evil are made vis-à-vis human behavior. The moral level of a person can be told by his moral character, and the moral level of a society reflects on all or most of the members'. Moral character is the relatively consistent personality traits or inclinations that an individual exhibits in behaving in accordance to the moral principles and norms of some specific society. According to the “four factors” theory of moral structure, moral character is made up of moral cognition, moral emotions, moral will and moral behavior. Measuring moral level can start with any one of “four factors” or the integrated factors.

The measurement of moral cognition began with Jean Piaget and Lawrence Kohlberg's clinical interview method [3]. Since then, researchers have developed various scales for the measurement of moral cognition [4]. With the development of implicit social cognition theory, researchers have applied implicit measurement methods to the measurement of moral cognition [5]–[6]. With a literature review about the recent moral measurement findings [7]–[8], we found the moral level judgment has been measured in various scales [9]–[10]. For example, Researchers measured the impact of moral strength on earnings management practices of accounting industry professionals [11]. But there has been little empirical research on factors affecting social morality levels, and existing research has only examined the impact of several demographic variables, individual characteristic variables and some of the environmental variables that may affect the level of morality [12]. These studies have laid a preliminary foundation for the construction of a more complete model of social morality, but more in-depth explorations of the various factors affecting moral behavior at the individual level, the familial level, the organizational level and the societal level are still needed; and the mechanisms of their actions also need to be clarified [13], so as to explore the emergence of turning individual's behavior into groups behavior.

Complexity thinking and the exploration of complexity have greatly deepened our understanding and recognition of the complexity of social systems. But at the same time, the increasingly evident complexity of social systems and their corresponding social scientific problems have also posed ever more new challenges to social scientific research methodology. As the complexity of social phenomena continues to increase, the corresponding research methodology which seeks to reveal and master the complexity has also continued to develop [14]–[15]. As a result, new research areas in social scientific computer-based modeling are also being created. In the field of sociology, the simulation method has been used primarily to study topics such as the appearance of social norms, how such norms react with individual social agents and influence their modes of behavior, peer influences, social changes, social dilemmas, complex networks, classroom interactions [16]–[20], and the interaction mechanism between group and individual.

Multi-agent simulation is a common computer simulation model in the social sciences [21]–[22], and it will likely become even more extensively applied in the future. ABM (agent-based modeling), which employs micro-level simulations to study macro-level phenomena, is also likely to become a key instrument for policy analysis in the future [23]. Due to differences in initial conditions and model interpretations, simulations can yield vastly different results. But with the aid of the NetLogo shareware platform [24], multiple iterations using different parameters and experimental conditions can be conducted with ease. For this reason, new research results based on the NetLogo platform are appearing with regularity [25].

However, the extant literature on morality research using the NetLogo platform has been conducted primarily among college students. In the world outside of the college campus, what kind of incentives would be the most effective at improving social morality? To what degree are these incentives required for achieving their desired effects? What are the conditions necessary for the maintenance of a superior level of social morality? To date, such questions have never received any quantitative answers. Most previous studies have approached these problems qualitatively on a macro level from the perspectives of society and culture, economic growth, public service, and other related issues. Attempts to measure social morality from a micro-level perspective through individual moral behavior have been few and far between. Through a set of computer-based experiments conducted on the NetLogo platform, this study investigates the relationship between moral and immoral behaviors from the perspective of individual moral behavior, and seeks to obtain precise measurements of social morality levels with the aid of computer simulations. The measurement of aggregate characteristics through individual behavior patterns in a complex system presents a novel approach for tackling similar research problems.

## Method Principle

### The conception of agent-based model

So far, there is no unified definition to describe the term “agent”. Generally speaking, any entity with independent thinking ability and interacting capability with the environment can be labeled abstractedly as agent. Agent also refers to a computing entities in a specific environment, and can continue to function independently, with resident, reactivity, social, initiative and other characteristics [26].

ABM is a computer model used to simulate behaviors of the independent individual or group (Agent or agents), or the individual interactions so as to explore the individual impact on the whole. This model can generate complex actions by setting up simple rules.

ABM is a computer simulation technique. This simulation can be used in situations where (1) it allows the researcher to understand key variables and illuminate avenues for future research; or (2) it is difficult to collect real-life data on particular phenomena; or (3) it is hard to dissociate inter-related or confounding factors within the researcher studying [27].

During the evaluation or judgment of the moral status, we put our attentions on the implementations of the moral codes which are shown through the individual actions. Each individual is given an action rule, and we let them move in the social environment and observe the characteristics shown by individual interaction. ABM is particularly suitable for studying this kind of moral phenomenon.

### NetLogo software

NetLogo is a programmable modeling environment for simulating natural and social phenomena. It was authored by Uri Wilensky in 1999 and has been in continuous development since then at the Center for Connected Learning and Computer-Based Modeling. NetLogo is particularly well suited for modeling complex systems developing over time. Modelers can give instructions to hundreds or thousands of “agents” all operating independently. This makes it possible to explore the connection between the micro-level actions of individuals and the macro-level patterns that emerge from their interaction [25].

NetLogo is a two-dimensional world made up agents. Agents carry out their own activity, all simultaneously, and include patches, turtles and observer.

**Figure 1:**
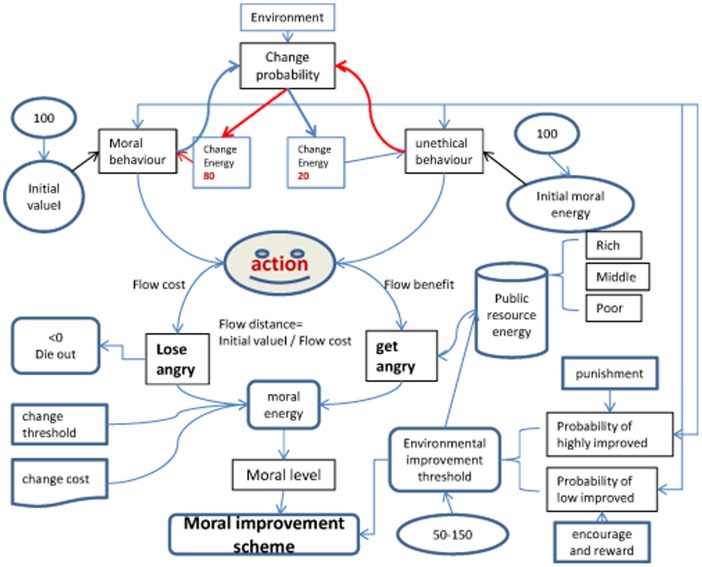
Moral computing simulation flowchart. This figure shows the basic principle of social moral level by quantitative analysis. As a starting point, we divide social agents into moral and immoral actors, and given their initial energy value. They move to gain or lose energy in the social environment. Public resources, agent energy value accord to set rules for interaction. Finally, we can get the level of social morality.

Patches are a static background which form a grid, don't move and have integer coordinates; turtles move on top of the patches, not necessarily in their center and have decimal coordinates and orientation and have different forms in different models, such as person, cattle, triangle, etc.; observer can create new turtles, and can have read/write access to all the agents and variables. Modelers can set the properties or rules of agents through NetLogo commands, in order to achieve a variety of simulation or emulation. Netlogo is a free software; and one can download the latest version software from the web site of NetLogo homepage [28]. The NetLogo interface of the simulation model is shown in Figure 1.

### Assumptions

To facilitate the computer simulation of social morality, we assume that:

1. Morality represents a society's positive value orientation, providing a basis for the assessment of the appropriateness of social behavior. Moral behavior in our model is not subject to regional and cultural influences. The magnitude of the values of moral norms is not considered, nor is the presence of moral tendencies or motivations.
2. Morality level (indicating the intensity of moral behavior) is indicated by the level of moral energy. All actions in the model carry some amount of moral energy. Moral actors have high levels of moral energy, and immoral actors have low levels of moral energy.
3. We will only consider agents engaged in moral and immoral actions. Other scenarios will not be considered for the time being.
4. Moral and immoral agents are mutually convertible under certain conditions. Specifically, agents are convertible where they are surrounded by sufficient neighbors of the opposite type; furthermore, the energy value required for a moral agent to convert to an immoral one is low, while the energy needed for the conversion of an immoral agent to a moral one is high.
5. Regarding the cost of moral behavior: Assume that people are characterized by limited rationality [29], so that they maximize their subjective utility when choosing a course of action. Let x denote the degree to which some action deviates from moral norms; and let p (x) denote the effect of that action, and R (x) denote the benefits from that action. Assume also that R (x) is an increasing function of x – in other words, the greater the deviation from moral norms, the greater the private benefits to an agent. Let C (x) denote the cost of the action, which comprises the following two parts: (1) The moral cost CM (x), which is an increasing function of x, so that the greater the behavioral deviation from moral norms, the greater the associated moral cost; and (2) The responsibility cost CD(x), which is a decreasing function of x, so that the greater the behavioral deviation from moral norms, the lower the responsibility cost. Thus:
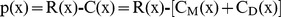
When p (x)>0, the associate action may be obtained. If multiple options are available, action x0 shall be chosen such that p (x) is maximized when x = x0. Among the various factors, CM (x) requires the greatest attention, as it can increase rapidly at the moment of a behavioral conversion. In addition, CM (x) includes two components: (1) The cost of autonomy CM0, which does not vary with x but is different from individual to individual. The higher the level of moral attainment, the higher CM0 would be; (2) The cost of heteronomy, which is an expected value. Let P (x) denote the probability that action x would be suppressed by others, and let u denote the resultant loss of utility, then CMV(x) = uP(x). Thus we have:
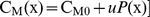

6. Existing social conditions shall remain relatively stable. Drastic changes brought on by regime changes are precluded.

### Model Description

Moral behavior is the product of social development. All social individuals are subject to the inculcation of the moral values of society from birth. The morality of a society exerts its influence on individual morality through an array of channels including the family, schools, and the social environment at large, although the degree of influence differs from individual to individual. The main factors that influence the level of social morality include self-discipline, social supervision, legal restrictions and the moral environment prevalent in a society, etc. The combined effect of all these factors determines the state or level of social morality, which is represented as the level of moral energy in the model. The state of social morality is therefore regulated by the value of moral energy in the model.

**Figure 2:**
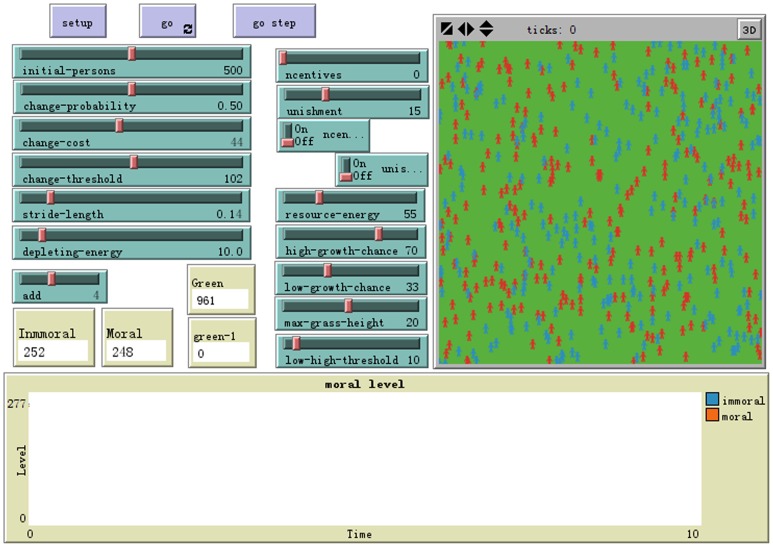
NetLogo interface of the simulation mode. NetLogo is a multi-agent programmable modeling environment. Here is the simulation interface that was developed by authors. This model includes the command area, parameters area, chart area and image area. Command area is responsible for the control of initialization parameters and operating procedures. Parameters area allows to dynamically adjust the parameters. The chart area display the simulation results. Image area can be dynamically displays the status of agents.

The assumptions and principles discussed above are summarized in the simulation flowchart presented in Figure 2.

As a starting point, we divide social agents (individual actors) into moral and immoral actors. All agents are endowed with an initial moral energy value regardless of type. Social agents move about their social environment with some specific rate of mobility, expending their own moral energy while obtaining energy from available public resources. The gains and losses in moral energy are calculated based on the associated moral costs. When an agent has exhausted all of its moral energy, the agent expires. Public resources will maintain the social morality level within a certain interval to prevent a complete collapse. When the energy value of public resources has become too low, improvements will be effected by punishing immoral behavior and rewarding moral behavior. When public energy has become too high, appropriate decreases to incentives will be made to allow for the emergence of new immoral behavior. The moral behaviors of social agents are mutually convertible. Conversion is regulated by the following three parameters: the cost of conversion, the threshold of conversion, and the probability of conversion.

### Selection of Parameters

There are many variables related to moral status. Morality consists of moral cognition, moral emotion, moral will and moral behavior, and each component can be subdivided into several sub-components. For instance, moral cognition can be subdivided into moral understanding, moral judgment and moral reasoning. There are many other factors influencing the moral level, such as the individual educational background, IQ, personality traits, social rewards and punishment and so on. Thus, all factors can be subdivided into several sub-factors. If we start working with variable selection by following this train of thought, the selection will go endless. This paper tries to use new method, and in order to test and verify the adaptability of the methods; the variable should be more typical and general. Therefore, this paper sets variables based on the synthetical status of individual moral situation, and named it as “moral energy”. The main variable is set according to the model description which is mentioned above. Other parameter design is derived in part from the cooperation model presented in notes [22] and [30].

The main parameters used in the model are listed in Table 1. The initial values are selected based upon past research experience and expert suggestions. The main purpose of setting initial values is to reduce the computational iterations needed in the interest of efficiency.

**Table 1:** Model Parameters and Initial Values.

Variable name	Code	Comments	Initial value
Moral agent	moral-agent		500
Immoral agent	immoral-agent		500
Initial moral energy	initial-energy	Both moral and immoral agents are endowed with identical initial energy values	50
Mobility rate	stride—length	During each unit of time in the simulation, each agent may move a certain distance in order to obtain energy and project influence. An increase in the range of an agent's influence can be interpreted as an increase in the scale of its behavior and the amount of energy generated. The rate of movement ranges between 0–0.3 per time unit.	0.08
Gains from mobility (energy of public resources)	Resource energy	Mobility allows an agent to obtain energy from public resources. This amounts to the agent's gains from mobility. As long as an agent lands at a location where public resources are available, it may obtain energy from those resources. The energy from public resources ranges from 0 to 200.	51
Cost of mobility	Depleting energy	Although mobility allows an agent to obtain new energy, the agent must also expend a certain amount of energy to execute the movement. This amounts to the cost of mobility. When an agent's energy level is reduced to 0, the agent is eliminated. The cost of mobility ranges between 0—99.	6
Cost of conversion	change cost	Whenever an agent is converted to an opposite type, a certain amount of energy must be expended. This value represents the energy cost of conversion. The cost of conversion ranges between 0—99	54
Threshold of conversion	change threshold	When an agent's moral energy reaches a certain value, it would acquire the opportunity to convert to the opposite type. This value represents the minimum energy necessary for an agent to obtain a conversion. This threshold value ranges between 0—100	80
Threshold of environmental improvement	low-high-threshold	This value ranges between 0 and 99. This is a threshold value which determines the level of resource regeneration. Above this threshold, resource regeneration proceeds according to the “probability of high improvement;” below this threshold, resource regeneration proceeds according to the “probability of low improvement.”	9
Probability of high improvement	high-growth-chance	Refers to the probability, expressed in percentage points, that the resource environment may improve when above the improvement threshold. The lower this value, the smaller the difference between moral and immoral behaviors.	77
Probability of low improvement	low-growth-chance	Refers to the probability, expressed in percentage points, that the environment may improve when below the improvement threshold. The greater this value, the smaller the difference between moral and immoral behavior.	30
Maximum energy of public resources	max-energy	Sets the maximum energy obtainable from social resources.	
Probability of conversion	Change probability	Probability of conversion between moral and immoral behavior	0.5
Degree of social incentives	Social incentives degree	The amount of incentive (expressed as an energy value) awarded by the social environment in response to a moral action. This value reflects the level of support rendered to moral behavior by the social environment. In the model, this is controlled through an on-off switch.	0
Degree of social punishment	Social punishment degree	The amount of punishment (expressed as an energy value) administered by the social environment in response to an immoral action. This value reflects society's sanction against immoral behavior, and is controlled through a switch in the model. Morality acts as a soft constraint imposed by society, whereas the law acts as a hard constraint imposed by the state. If legal norms provide the skeletal framework encompassing the social domain, then the empty spaces within the framework constitute the domain regulated by moral norms. Social sanctions play a role similar to the law.	0
Morality level	Moral-level	The ratio of moral agents to immoral agents.	

## Experiment and Results

### Experimental design

In order to better understand changes in the level of social morality and identify those factors and mechanisms which may affect and control the level of social morality, this study relies primarily on univariate and multivariate crossover simulations. In a univariate simulation, we modify the value of a single variable while holding all other variables constant, and examine its effect on the experimental outcome. In a multivariate crossover simulation, two or more variables may be modified simultaneously in order to examine their effects on the experimental outcome.

### Univariate simulations

The simulations here were conducted under univariate controls. In other words, the results were obtained by adjusting one particular variable while holding all other variables constant at their initial values.

**Figure 3:**
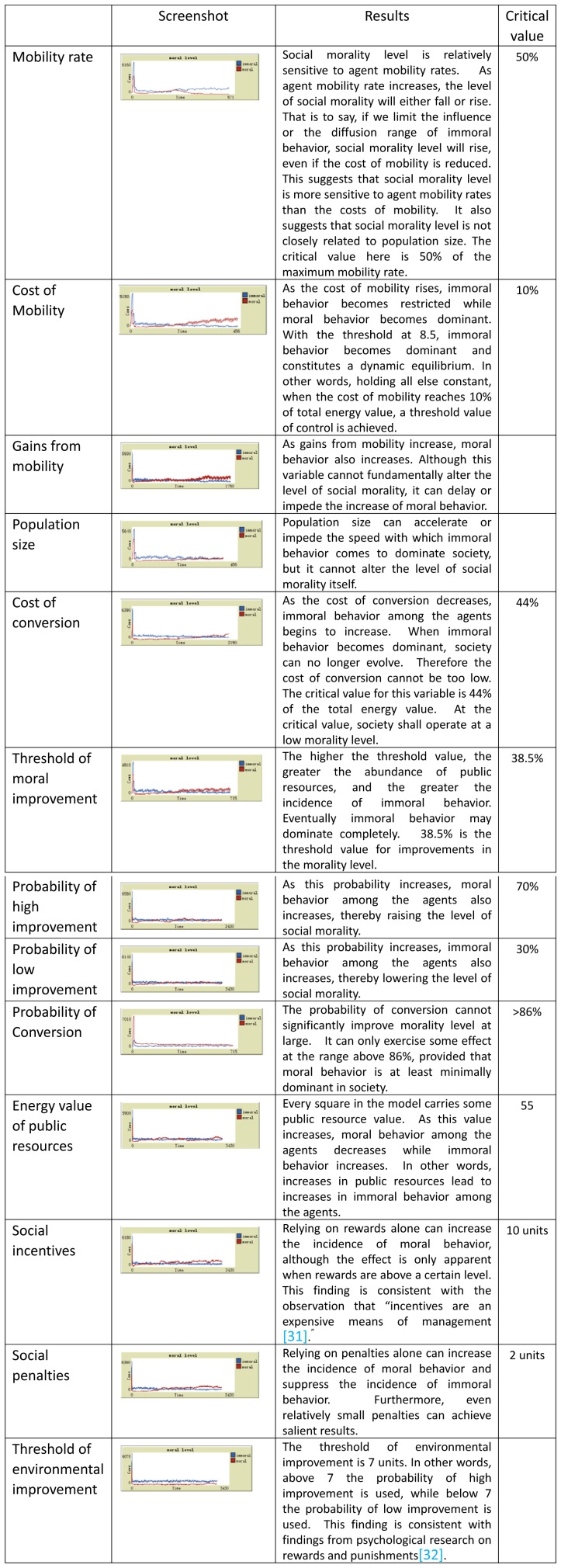
Simulation results with univariate controls. In this type of analysis, we can observe the effects of the changes in the overall social moral level by adjustments of one variable parameter value. Critical state parameter values are found after repeated experiments. Some results consistent with other research conclusions. Such as incentives are an expensive means of management [31] and psychological research on rewards and punishments [32].

The experiment process was conducted under controlling conditions. The section carried out the experiment by changing single variable value; that means other parameter values were not changed by operator. We observed the effects of the changes in the overall social moral level by adjustments of one variable parameter value. Critical state parameter values were found after repeated experiments, and the values impacted the change of social moral status and trends by slight changes. The results are presented in Figure 3.

### Multivariate simulations

**Figure 4:**
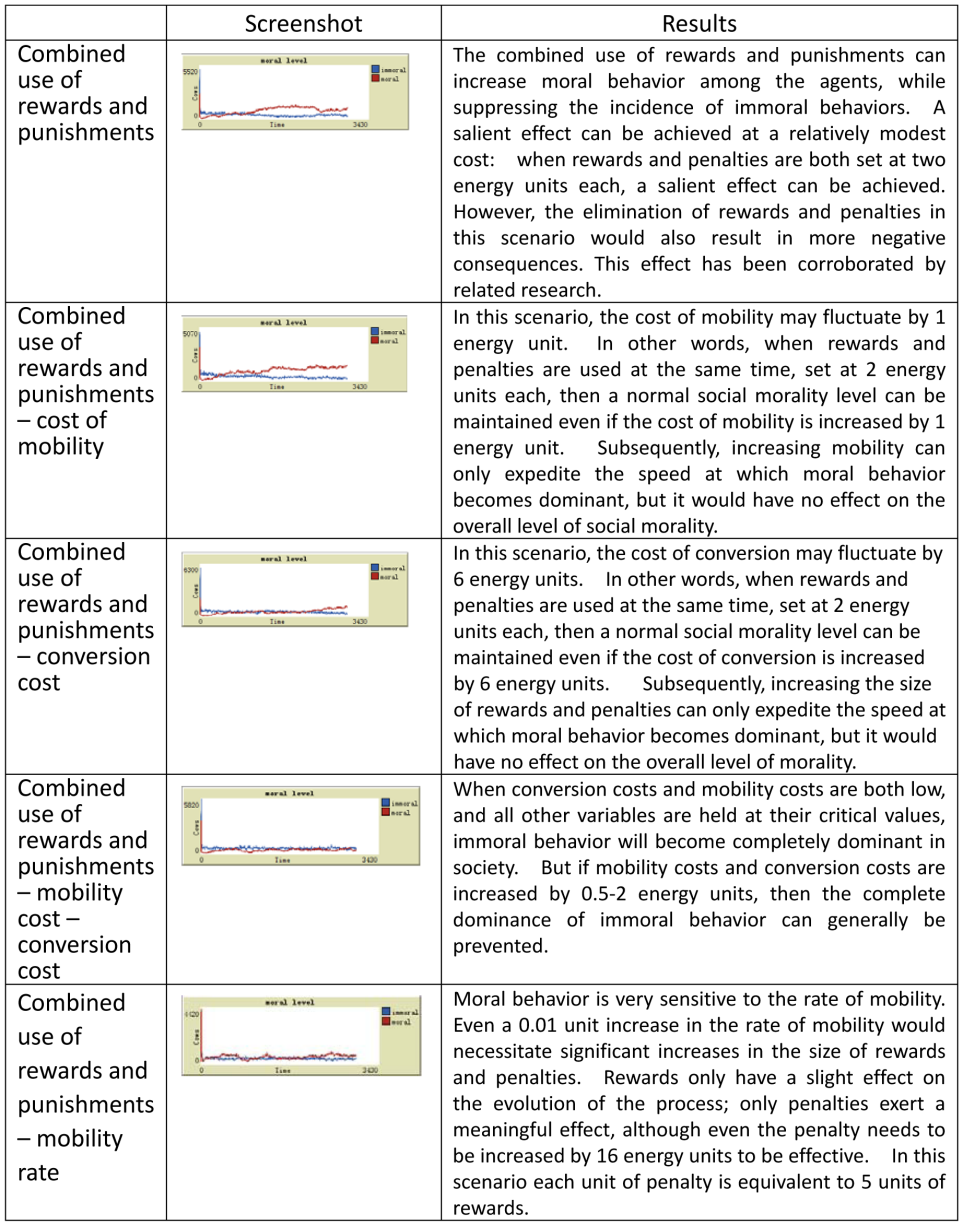
Simulation results with multivariate controls. In this type of analysis, we can seek to observe changes in the overall level of social morality while modifying two or more variables simultaneously.

Multivariate analysis is conducted using the critical values obtained from the univariate analyses discussed above. In this type of analysis, we seek to observe changes in the overall level of social morality while modifying two or more variables simultaneously. The results from the simulations are presented in Figure 4.

### Highlighted finding results

The results of the experiment have yielded the following highlighted findings:

1. Population size may accelerate or impede the speed with which immoral behavior comes to dominate society as a whole, but it has no effect on the final level of social morality.
2. The relative impact of rewards and punishments following the “5∶1 rewards-to-punishment rule,” which is to say that 5 units of rewards have the same effect as 1 unit of punishment, is consistent with the observation that “rewards are an expensive means of management.”
3. The abundance of public resources is inversely related to the level of social morality. In other words, immoral behavior tends to increase quickly when public resources are abundant, and decreases when they are not.
4. Moral behavior is highly sensitive to the level of population mobility – a small amount of increase in the latter would require large adjustments in rewards and punishments. Rewards only have a slight effect on the evolution of the process; only punishment exerts a meaningful effect, although even punishment needs to be increased by 16 energy units to be effective.
5. When rewards and punishment are used in combination, with both set at 2 energy units, moral behavior can remain dominant even when mobility cost is increased by 1 unit.
6. Holding all else constant, when the cost of mobility reaches 10% of the total energy level, it begins to manifest an inhibiting effect on immoral behavior. From this, the “1/10 moral cost rule” follows.

## Discussion

### On Methodology

Agent can help people use computer simulation to do experiments, and to obtain results which can hardly be obtained in a traditional manner. Through obtained variable data from computer assignment (strictly controlled “inconstant” and “constant” of parameters) we manage to build a strict experimental environment which is similar to natural science. The experiment demonstrates the flow of specific operation processes obtaining results from the simulation system offers references for decision-making and explores the general law of system evolution. In the specific case of empirical study, more targeted and guided decision-making reference can be collected by input data, which can be collected by field survey (e.g. by questionnaire, etc.) into the simulation platform. The model prototype and the experimental results above, especially the highlighted finding results, basically achieved the requirements which can offer some reference suggestions for decision making.

There are some drawbacks of traditional moral quantitative research, as well as in statistics itself. Also, there is no 100% accuracy in sample deduction [33]. In moral study, an integrated approach is required [34]–[35]. The research method adopted in this paper only takes a few minutes to get results. It also has irreplaceable advantages when compared to other methods like using questionnaire, statistics, interview method, etc.

There is much qualitative data and semi-structured data in social simulation, and it is difficult to get real evidence, and also impossible to use the method of completely quantitative and empirical validation to test if the “model can represent the real world”. Therefore, qualitative validation method is applied in this paper. If the input - output was in line with common sense, the model was effective; otherwise, it is invalid. The experimental process and results above conform to the qualitative knowledge in the field of ethics.

### Main suggestions

According to the experimental results discussed above, we put forward the following recommendations in response to moral decline and rampant social apathy:

1. We recommend that the government should launch an extensive moral education campaign, in conjunction with a system of heavy penalties and modest rewards, so as to limit the spread and influence of immoral behaviors, and to create a social atmosphere universally hostile toward immorality. In particular, a 10% “moral cost” is found to be the threshold value for the suppression of immoral behaviors. In other words, if the penalty imposed amounts to 10% of total income, then immoral behaviors will begin to recede.
2. By controlling the expansion of public resources, we can accelerate the formation of social morality. Holding all else constant, the threshold value for any increase in public resources is roughly a quarter of the total resource value, or 55 energy units. When this threshold is exceeded, immoral behavior will begin to increase.
3. In the employment of rewards and punishments, meaningful effects can only be achieved through an emphasis on punishment in conjunction with the appropriate use of rewards. From the perspective of social psychology, the main method of suppressing misconduct or immoral behavior should be through increasing the cost of immoral behavior. Only by reducing the gains obtainable from public resources through misconduct can a corrective effect be quickly achieved. Rewarding moral behavior through an increase in public resources clearly does not produce as salient a psychological effect as punishment. The profound social apathy prevalent in China today is no doubt closely related to the low psychic costs associated with various immoral behaviors [36].

## Conclusions

Morality has again become an important focus of research in different scientific disciplines [37]. This study demonstrates that accurate measurements of morality levels are possible with the aid of ABM models, guided by the principles of complexity science [38]. We believe it offers a range of potential benefits [39]. NetLogo makes it possible for more and more researchers without a professional programming background to explore their theoretical conceptions dynamically; and it also enables them to predict and simulate possible problems that may arise in real life before or after the empirical study, so that they may refine their empirical research designs.

It is discovered that some highlighted findings results derived from the part of the univariate and multivariate experiment results coincide with our qualitative knowledge, and they can be used as the basis for aiding decision-making in policy. This experimental research method, which is quantitive, repeatable and optimizable, has irreplaceable advantages over traditional methods.

This article represents the author's preliminary research findings. Future research shall further investigate the effect on morality of different individual moral levels and motivations, as well as the impact of different localities and cultures, so that the validity and accuracy of morality measurements can be further refined.
